# Wearable Technology in Diving: A Review of Heart Rate and Oxygen Saturation Monitoring for Enhanced Safety and Performance

**DOI:** 10.3390/healthcare13182346

**Published:** 2025-09-18

**Authors:** Tae Sung Park, Min-Gyu Kim, Jong-Hwan Park, Jeong-Min Hong, Dowon Lee, In Ho Han, Myung-Jun Shin

**Affiliations:** 1 Department of Convergence Medical Institute of Technology, Pusan National University Hospital, Busan 49241, Republic of Korea; tsbark@naver.com (T.S.P.);; 2 Department of Biomedical Research Institute, Pusan National University Hospital, Busan 49241, Republic of Korea; 3 Human-Robot Interaction Research Center, Korea Institute of Robotics and Technology Convergence, Pohang 37666, Republic of Korea; 4 Department of Convergence Medicine, Pusan National University School of Medicine, Yangsan 50612, Republic of Korea; 5 Department of Clinical Bio-Convergence, Graduate School of Convergence in Biomedical Science, Pusan National University School of Medicine, Yangsan 50612, Republic of Korea; 6 Department of Anesthesia and Pain Medicine, Pusan National University Hospital and Pusan National University School of Medicine, Busan 49241, Republic of Korea; 7 Department of Neurosurgery, Pusan National University and Pusan National University School of Medicine, Busan 49241, Republic of Korea; 8 Department of Rehabilitation Medicine, Pusan National University Hospital and Pusan National University School of Medicine, Busan 49241, Republic of Korea

**Keywords:** diving, heart rate, oxygen saturation, physiological monitoring, wearable devices

## Abstract

Monitoring heart rate (HR) and peripheral oxygen saturation (SpO_2_) in underwater environments has gained increasing importance due to the expanding popularity of diving activities such as SCUBA diving, freediving, and professional underwater operations. These physiological parameters are critical indicators for detecting adaptive responses and early signs of physiological distress caused by environmental stressors like elevated ambient pressure, hypoxia, cold temperatures, and psychological stress. Although recent advances in wearable sensor technologies offer new opportunities for real-time physiological monitoring underwater, significant limitations persist due to issues such as signal interference, cold-induced vasoconstriction, sensor durability, and the complexity of reliably measuring these parameters in dynamic underwater conditions. Evidence shows HR can fall by more than 50% in freedivers and SpO_2_ may decline to below 50% during repeated dives, with proposed depth-specific thresholds (e.g., <98.5% at 30 m) serving as early warning levels. This review synthesizes current knowledge on the cardiovascular and oxygenation responses observed during diving, explores the technological challenges associated with underwater HR and SpO_2_ monitoring, and discusses future directions, including the integration of multisensor platforms and predictive analytics to enhance diver safety and physiological monitoring capabilities. Addressing these technological and methodological gaps holds the potential to substantially improve safety standards and expand the clinical applicability of underwater physiological monitoring systems.

## 1. Introduction

Recreational and professional diving activities, including SCUBA diving, freediving, and military or industrial underwater operations, have expanded in scope and popularity. These activities expose the human body to extreme environmental conditions such as high ambient pressure, cold temperatures, hypoxia, and psychological stress [1]. In addition, divers may encounter nitrogen narcosis, a depth-related condition caused by elevated partial pressures of nitrogen, which can impair cognition, judgment, and motor control [2,3]. Consequently, understanding the physiological responses to underwater environments has become a critical area of interest not only in sports science but also in clinical safety management. In particular, heart rate (HR) and oxygen saturation (SpO_2_) are key physiological indicators that reflect the body’s adaptive responses to such conditions [1,4]. These parameters play an essential role in predicting fatigue, detecting hypoxic states, and preventing life-threatening conditions such as decompression sickness (DCS). However, current monitoring practices and standard dive computers predominantly track environmental variables (e.g., depth, time, ascent rate, gas mixture) rather than a diver’s real-time physiological status. Moreover, conventional HR/SpO_2_ devices suffer from motion artifacts, water ingress, cold-induced peripheral vasoconstriction, and pressure effects that degrade signal quality in open water [5,6,7].

Decompression sickness, a condition arising from an inadequate elimination of inert gases during ascent, is associated with a wide range of symptoms, from mild fatigue and joint pain to severe neurological deficits [8]. Fluctuations in HR and SpO_2_ may serve as early warning signs of physiological instability during diving, especially when combined with environmental challenges like cold exposure or repeated dives [1,4]. In addition, inter-individual characteristics such as age, physical fitness, and chronic health conditions can significantly influence these physiological responses, potentially altering autonomic regulation and complicating the interpretation of underwater monitoring data. Another important factor is CO_2_ retention, also referred to as hypercapnia, which increases ventilatory drive and alters autonomic control. When combined with hyperoxia at depth, hypercapnia can exacerbate the risk of hypoxic events and further destabilize cardiopulmonary responses during diving [9,10].

Despite the increasing use of wearable devices in sports and health monitoring, real-time tracking of HR and SpO_2_ during actual open-water dives remains limited [1,11]. Most studies have either relied on retrospective data analysis or laboratory-based simulations that do not fully replicate real-world underwater conditions [11,12]. Furthermore, inconsistencies in observed HR patterns, ranging from significant bradycardia to stress-induced tachycardia, suggest that multiple variables such as dive depth, water temperature, physical workload, and diver experience influence the autonomic response to diving [11]. Existing wearable systems adapted for diving generally include electrocardiogram (ECG)-based chest straps with waterproof electrodes, forehead- or temple-mounted pulse oximeters, and wrist-worn photoplethysmography (PPG) devices [13]. These devices are non-invasive and designed for continuous physiological monitoring under immersion, although their performance can be affected by environmental factors such as low temperature, body movement, and increased water pressure. Beyond these commercially available devices, recent research has proposed advanced systems such as real-time ECG monitoring with automated alert functions [5], customized diving computers linked to textile-based wearable sensors that capture respiration and body temperature [6], and integrated diver masks equipped with augmented reality (AR) and head-up displays (HUDs) that present biometric and environmental data in real time [7]. These emerging technologies highlight ongoing efforts to overcome the limitations of conventional devices and to enhance diver safety through multisensor integration and intelligent risk detection.

Given the physiological significance of HR and SpO_2_, and the lack of standardized monitoring practices during diving, this review aims to synthesize current knowledge on underwater cardiovascular and oxygenation responses. Through a narrative overview of existing literature, we identify key trends and methodological challenges in measuring these vital signs under real underwater conditions. While prior reviews have often focused on isolated physiological mechanisms or single-sensor applications in controlled environments, our approach integrates findings across multiple domains. Specifically, we emphasize HR–SpO_2_ coupling, summarize depth-specific SpO_2_ thresholds, and explore the potential of multi-sensor fusion with AI-assisted early detection. By framing these advances in relation to both recreational and professional diving contexts, including occupational safety, military operations, and clinical rehabilitation, this review highlights the broader relevance of underwater wearable monitoring and lays the groundwork for future research and improved diver safety.

To guide this narrative review, a targeted literature search was conducted using the Google Scholar database. Keywords included “diving physiology,” “underwater heart rate,” and “SpO_2_ in diving.” Studies published from 1980 to 2024 were reviewed, emphasizing research conducted in open-water conditions, physiological monitoring methodologies, and clinical relevance.

## 2. Physiological Responses to Underwater Environments

### 2.1. Environmental Stressors During Diving

Diving environments present a combination of extreme physical and psychological challenges that influence human physiology. Key environmental stressors include increased ambient pressure, cold water temperature, reduced oxygen availability (hypoxia), and psychological stress related to depth, darkness, and isolation [1,14]. Exposure to hyperbaric conditions alters gas exchange dynamics and increases the partial pressure of oxygen and inert gases, which may have both protective and harmful effects depending on dive duration and depth [14]. Cold temperatures contribute to peripheral vasoconstriction and increase cardiovascular strain [15,16], while mental stress can activate sympathetic responses that counteract natural diving adaptations [17,18]. Collectively, these stressors necessitate a suite of rapid and integrated physiological responses to preserve homeostasis during immersion. Importantly, these environmental stressors do not act in isolation but often interact synergistically, amplifying physiological burden. For instance, cold-induced vasoconstriction may worsen hypoxic responses by impairing peripheral oxygen delivery, and psychological stress can modulate autonomic responses, potentially altering protective physiological mechanisms such as the mammalian diving reflex (MDR).

### 2.2. The Human Dive Reflex

One of the most prominent physiological adaptations to immersion is the MDR, a conserved autonomic response characterized by bradycardia, peripheral vasoconstriction, and blood redistribution toward vital organs [19,20]. This reflex is typically triggered by facial contact with cold water and breath-holding (apnea), aiming to optimize oxygen conservation for essential organs such as the heart and brain. Although more pronounced in trained freedivers, MDR can also be observed in recreational SCUBA divers, particularly under stressful or cold conditions [19,20,21]. Bradycardia serves to reduce myocardial oxygen demand [19,20], whereas vasoconstriction helps maintain arterial pressure despite central volume shifts [20,22]. However, individual variability in MDR expression has been reported, influenced by factors such as fitness level, experience, and psychological state [21,22].

### 2.3. Autonomic Nervous System Regulation

The autonomic nervous system (ANS) plays a central role in coordinating cardiovascular responses during diving. Immersion typically activates both branches of the ANS: parasympathetic dominance promotes bradycardia, while sympathetic activation may occur concurrently in response to cold exposure or psychological stress [22]. This dual activation results in complex cardiovascular outcomes, with inter-individual differences in HR responses ranging from pronounced bradycardia to episodes of stress-induced tachycardia [22,23]. Environmental stimuli such as water temperature, visibility, and exertion can dynamically shift the ANS balance, influencing cardiac output, vascular tone, and respiratory drive during immersion [23]. The coexistence of parasympathetic and sympathetic activation during diving, often referred to as autonomic conflict, may predispose certain individuals to arrhythmias, especially under cold water or emotional stress conditions. This autonomic conflict has been suggested as a potential contributing factor in some unexplained diving incidents.

## 3. Heart Rate and SpO_2_ as Vital Indicators in Diving

### 3.1. Physiological Significance of Heart Rate Changes

**Figure 1:**
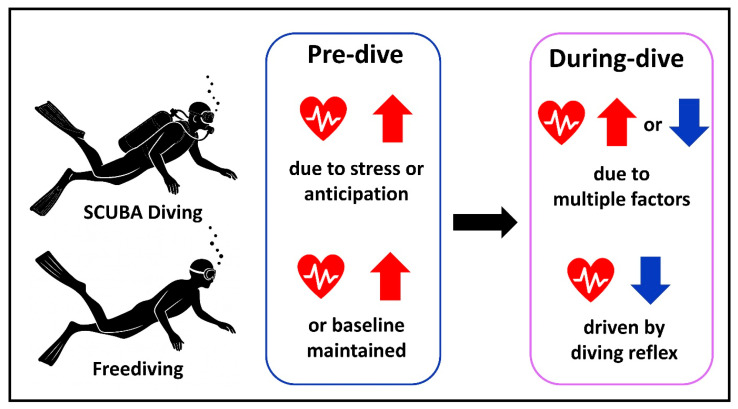
Heart Rate Response Before and During Diving: Comparison Between SCUBA and Freediving. Pre-dive HR may increase due to psychological stress or anticipation in both modalities, although trained freedivers may exhibit lower baseline levels. During diving, SCUBA divers may show variable HR responses depending on physical exertion and environmental stress, while freedivers typically demonstrate bradycardia driven by the mammalian diving reflex.

HR plays a central role in the body’s adaptive response to immersion and breath-hold diving. One of the hallmark responses is diving bradycardia, primarily mediated by parasympathetic activation through the MDR, which serves to reduce myocardial oxygen consumption and redirect blood flow toward vital organs such as the brain and heart [19,22]. This reflex is particularly pronounced in trained freedivers but can also be observed in SCUBA divers exposed to cold or psychological stress [24,25]. While bradycardia is often interpreted as a protective adaptation, the degree of HR reduction varies significantly across individuals, with some exhibiting minimal changes or even transient tachycardia under stressful conditions [26]. These HR responses vary widely depending on individual fitness, diving modality, and environmental conditions. In recreational freedivers, HR reductions of 31–45% from baseline have been reported during repeated dives, with some individuals exhibiting decreases of up to 58–67% and minimum HR values as low as 28 bpm [27,28]. Studies involving elite competitive freedivers have shown average reductions of approximately 46%, regardless of depth [13]. Interestingly, shallow dives (e.g., ~17–19 m) tend to elicit greater HR decreases than deeper dives [13,28], possibly due to reduced muscular effort during passive descent. In contrast, SCUBA divers generally experience more modest HR reductions, averaging around 21% (range: 5–39%) [1]. Notably, under extreme conditions such as near-freezing water, some divers exhibit paradoxical HR increases, likely mediated by heightened sympathetic activation [29]. These variations are influenced by a range of factors, including water temperature, mental preparedness, physical fitness, and the level of diving experience [24,30]. In some cases, exposure to cold, increased physical effort, or acute psychological stress may predominantly activate the sympathetic nervous system, leading to tachycardia rather than bradycardia [29,31]. This response is more frequently observed among novice or less-adapted divers. Understanding HR dynamics during diving is essential not only for optimizing performance in recreational and competitive diving but also for early detection of physiological instability and diver fatigue. A graphical comparison of HR patterns observed in SCUBA and freediving is presented in Figure 1. It illustrates anticipatory HR increases before immersion and divergent HR trends during diving, highlighting the contrasting autonomic responses characteristic of each diving modality.

Given these individual variations, establishing personalized baseline HR values may be critical for effective physiological monitoring. For instance, elevated pre-dive HR could reflect increased sympathetic activation or psychological stress, indicating a higher risk for dysregulation. By collecting HR profiles during stable, uneventful dives, each diver’s normal physiological range can be defined.

### 3.2. Patterns of SpO_2_ Desaturation and Recovery

SpO_2_ provides a noninvasive index of peripheral oxygen availability and has been widely used to monitor hypoxic stress in both clinical and environmental settings [32,33]. During breath-hold diving or prolonged underwater activity, a gradual decrease in SpO_2_ can be observed due to reduced pulmonary ventilation and increased oxygen extraction by active tissues [13]. This desaturation is more pronounced in freedivers, particularly during repetitive dives with insufficient surface intervals, which can lead to cumulative hypoxia [13]. In SCUBA divers, although continuous gas supply generally prevents severe hypoxemia, SpO_2_ monitoring remains clinically and operationally relevant. It can serve as an early indicator of equipment malfunctions (such as regulator failure or improper gas mixture), insufficient oxygenation due to depth-related factors, or early signs of oxygen toxicity in cases where high partial pressure of oxygen (PO_2_) is present, especially during technical or CCR (closed-circuit rebreather) dives [11,34]. In addition, deviations from expected SpO_2_ trends may reflect physiological strain, cold-induced vasoconstriction, or impaired recovery capacity after long or deep dives [22]. Cold water immersion reduces cutaneous perfusion, which may delay reoxygenation upon surfacing [35], and repetitive diving has been shown to prolong the time required for SpO_2_ recovery after resurfacing [27]. The rate and extent of SpO_2_ decline depend on several factors, including dive duration, water temperature, workload intensity, and pre-dive oxygen reserves [19,36]. In trained individuals, a more controlled and gradual desaturation pattern is often observed, reflecting physiological adaptations such as enhanced oxygen storage, slower metabolic rate, and tolerance to hypoxia [13]. In contrast, rapid or severe desaturation may indicate inadequate conditioning or excessive physiological demand [13]. Recovery of SpO_2_ typically occurs within seconds to minutes upon resurfacing and initiating breathing. However, the speed of reoxygenation may be delayed in cases of hypercapnia, cold-induced vasoconstriction, or compromised cardiopulmonary function [37]. Monitoring the pattern of SpO_2_ recovery can thus provide insight into the diver’s physiological resilience, ventilatory efficiency, and potential risk for hypoxia- or hyperoxia-related complications.

### 3.3. HR–SpO_2_ Coupling and Predictive Potential

Simultaneous assessment of HR and SpO_2_ provides a comprehensive view of cardiopulmonary adaptation to underwater environments. During immersion, a reduction in HR typically occurs alongside gradual SpO_2_ desaturation, reflecting activation of the mammalian diving response and redistribution of blood flow toward vital organs [19,22]. In trained divers, this coupling often shows a stable and gradual pattern in which bradycardia accompanies controlled desaturation, indicating effective oxygen conservation and autonomic balance [13,38]. In contrast, abnormal patterns such as insufficient HR reduction despite falling SpO_2_, or transient tachycardia combined with rapid desaturation, may signal physiological strain, inadequate conditioning, or early signs of equipment-related problems [25,39,40]. For example, such patterns have been associated with impending hypoxic blackout in freedivers and with regulator malfunction or CO_2_ retention in SCUBA divers.

In SCUBA divers, where a continuous supply of breathing gas generally maintains oxygenation, HR–SpO_2_ monitoring remains valuable for detecting unexpected events. Equipment malfunctions such as regulator failure or improper gas mixture can result in unanticipated SpO_2_ drops [41], and cold exposure or high physical workload can evoke sympathetic activation that elevates HR even when oxygen supply is stable [22]. Observing these combined signals could be helpful in detecting physiological instability or operational issues in both recreational skin scuba diving and technical diving scenarios. Building on this concept, recent developments in wearable sensors and machine learning techniques are being explored to enable real-time modeling of HR–SpO_2_ coupling, showing potential to provide predictive insights into hypoxic events, impending syncope, and oxygen toxicity in both freediving and SCUBA contexts [11,13].

## 4. Monitoring HR and SpO_2_ in Underwater Environments

### 4.1. Technological Approaches and Sensor Limitations

Monitoring HR and SpO_2_ in underwater environments requires specialized sensor systems that can operate reliably under high pressure, low temperature, and dynamic motion. Conventional medical-grade devices, such as fingertip pulse oximeters or chest-strap heart rate monitors, often experience signal distortion when submerged due to water infiltration, sensor movement, and changes in tissue perfusion [11,27]. Consequently, recent years have seen the development of wearable sensors specifically adapted for aquatic use [13]. A summary of representative studies evaluating these monitoring approaches is provided in Table 1.

One widely used approach for HR monitoring is the ECG-based chest strap, which employs waterproof electrodes and encapsulated transmitters to maintain signal integrity during immersion [29,44]. These devices, originally designed for swimming and triathlon training, have been adapted for diving by incorporating pressure-resistant casings and improved sealing to prevent corrosion and electrical interference [17,44]. Optical sensors based on PPG have also been explored, typically embedded in wrist-worn or forehead-mounted devices. However, PPG sensors are particularly sensitive to motion artifacts and reduced peripheral blood flow caused by cold-induced vasoconstriction, which can compromise signal quality underwater [45,46].

For SpO_2_ monitoring, specialized underwater pulse oximeters have been developed. These systems typically position infrared and red light emitters on thin skin regions such as the forehead, temple, or earlobe, where perfusion remains relatively stable during immersion [43]. Researchers have developed custom-made sensors designed for thin, well-perfused skin regions such as the forehead or temple, with silicone housings to minimize water intrusion and pressure effects [13,43]. Some prototypes now integrate both HR and SpO_2_ measurement in a single module, with data typically stored locally for post-dive analysis [13,43], although real-time transmission to surface units or data loggers will likely be necessary for future developments.

Despite recent advances, current methods remain limited. Even devices that perform well in laboratory settings are likely to experience degraded signal quality in open-water conditions due to factors such as pressure fluctuations, cold exposure, and movement. For example, pool-based evaluations of wearable ECG systems have reported that motion artifacts, electrode displacement, and unstable wireless connections reduced real-time detection accuracy to around 90% under controlled conditions [5]. Similarly, customized diving computers integrating textile-based respiration and temperature sensors showed acceptable performance at shallow depths but decreased accuracy at greater depths, indicating the influence of ambient pressure and cold exposure on signal fidelity [6]. Other studies have emphasized that diver equipment configuration and environmental stressors can further distort biosignals, underscoring the need for calibration and error-correction protocols [7]. In particular, cold water exposure induces peripheral vasoconstriction, which decreases blood flow in extremities and can delay or underestimate SpO_2_ values in fingertip pulse oximeters [43,47]. For this reason, forehead- or temple-mounted probes are often preferred, as they are less affected by vasoconstriction, though they remain vulnerable during prolonged immersion [43,47]. Moreover, sensor calibration under pressure and temperature variation has not been standardized. Validation studies have used waterproof housings pressure-tested to several hundred kilopascals and Bland–Altman analysis to confirm reliability compared with surface recordings [42], while pulse oximeters have been validated against arterial blood gas analysis [11,43]. However, such procedures are difficult to perform at depth [13], reinforcing the need for robust calibration protocols and algorithmic noise reduction to ensure reliable underwater monitoring. Power consumption and data transmission further illustrate critical limitations of current systems. Recent prototypes of underwater pulse oximeters have been designed with low-power processors and rechargeable lithium-ion batteries, emphasizing energy-efficient operation, yet systematic evaluations of battery autonomy during prolonged or deep dives remain scarce [5,43]. With respect to communication, Wi-Fi is unsuitable for submerged use, whereas optical fiber can enable stable data transfer but is impractical in most diving scenarios and has so far been limited to controlled experiments [13]. Consequently, most devices rely on local micro-SD storage with post-dive analysis [43]. Although real-time transmission to the surface has been proposed as a future goal, comprehensive evaluations of transmission reliability and latency are still lacking [7]. Issues such as waterproofing, long-term durability, and ergonomic design continue to affect usability, and PPG-based systems show inconsistent accuracy under cold-induced vasoconstriction [45]. To address these limitations, researchers are developing integrated multisensor platforms that combine HR and SpO_2_ with data from accelerometers, skin temperature sensors, and pressure sensors [7]. Although these approaches are still in experimental stages, they show promise for improving reliability and safety in both recreational and technical diving contexts. In our view, future progress in underwater physiological monitoring will require not only further integration of multiple sensing modalities into a single robust device but also reliable real-time data transmission to surface units, which will be essential for timely decision-making and enhanced diver safety [7].

### 4.2. HR Changes Under Hyperoxia at Depth

As a diver descends, ambient pressure increases by approximately 1 atmosphere (ATA) for every 10 m of seawater [48,49]. Since the partial pressure of inspired oxygen (PiO_2_) is directly proportional to ambient pressure and the oxygen fraction in the breathing gas, PiO_2_ increases significantly with depth [48]. For example, when breathing air (21% O_2_), PiO_2_ reaches approximately 1.6 ATA at a depth of around 66 m, which is the threshold commonly associated with central nervous system (CNS) oxygen toxicity [48]. The depth at which the oxygen toxicity threshold (1.6 ATA PiO_2_) is reached becomes shallower when enriched oxygen mixtures, such as Nitrox, are used, as shown in Table 2 [48,50].

The cardiovascular response to moderate hyperoxia below toxic thresholds is characterized by vagally mediated bradycardia and reduced sympathetic outflow. These changes are believed to lower myocardial oxygen demand and improve autonomic balance [51,52,53]. According to Bosco et al. (2018) [54], hyperoxic conditions at depth may attenuate ventilatory chemosensitivity and promote CO_2_ retention, thereby influencing autonomic regulation. Furthermore, they noted that elevated PO_2_ can modulate cardiopulmonary function by altering the balance of reactive oxygen species and antioxidant enzyme activity, potentially supporting enhanced physiological adaptation and vascular protection during diving.

While healthy individuals generally tolerate these effects without complications, divers with pre-existing cardiovascular conditions may be more vulnerable to adverse responses. The combination of elevated PiO_2_ and immersion-related stressors such as cold-induced vasoconstriction, immersion diuresis, and psychological stress may potentially increase susceptibility to arrhythmias or delay post-dive HR recovery, although further research is needed to clarify these risks. Although hyperoxia below toxic thresholds is typically well tolerated, a deeper understanding of the interactions between oxygen partial pressure, autonomic responses, and cardiovascular health remains essential for ensuring diver safety and for optimizing breathing gas strategies in technical diving.

### 4.3. Depth-Specific Interpretation of Oxygen Saturation

SpO_2_ is a widely used indicator of oxygenation status in terrestrial and clinical settings. However, its interpretation during SCUBA diving requires a context-specific approach. As depth increases, the PiO_2_ rises linearly with ambient pressure, leading to arterial oxygen partial pressures (PaO_2_) that far exceed surface-level values [55]. Under these conditions, the oxygen–hemoglobin dissociation curve reaches a plateau phase, where SpO_2_ is expected to remain close to 100% despite substantial fluctuations in PaO_2_.

This physiological characteristic suggests that conventional SpO_2_ thresholds, such as the clinical warning level of <95%, may be inadequate or misleading at depth. For example, a SpO_2_ reading of 95% at 30 m, where PiO_2_ is approximately 0.84 ATA, may indicate a significant deviation from the expected baseline and reflect early signs of gas supply irregularity, ventilatory inefficiency, or autonomic dysregulation [55]. Even small deviations from the saturation norm warrant attention due to the elevated oxygen availability under hyperbaric conditions [55].

Table 3 summarizes proposed SpO_2_ alert thresholds corresponding to depth under normobaric air breathing [28,55,56]. These thresholds are synthesized from previously published studies, and in areas where evidence was limited, provisional interpretations were added by the authors to serve as a starting point for discussion. They progressively tighten with increasing depth, reflecting the expectation of higher saturation baselines. Rather than being universal cut-offs, these values are intended as reference guidelines for wearable device algorithms, diver monitoring protocols, and emergency response systems, and should be interpreted in relation to other stressors such as cold exposure, workload, and gas mixture. A dynamic, depth-adjusted interpretation of SpO_2_ can enhance diver safety by facilitating early detection of physiological or operational anomalies before clinical symptoms manifest.

## 5. Clinical and Safety Implications

### 5.1. Early Detection of Physiological Warning Signs

Continuous monitoring of HR and SpO_2_ in underwater environments provides important clinical and operational benefits, particularly for detecting early signs of physiological strain. As previously discussed, specific HR and SpO_2_ response patterns may indicate developing autonomic imbalance, hypoxia, or equipment-related anomalies before clear symptoms appear. In practical settings, real-time deviations from a diver’s baseline, such as unexpected tachycardia, insufficient bradycardia, or delayed SpO_2_ recovery, can suggest excessive physical workload, psychological stress, or impaired ventilation. SpO_2_ primarily reflects oxygen availability and ventilation–perfusion balance; reductions in SpO_2_ may indicate emerging hypoxic conditions [11]. Likewise, abnormal HR patterns can reflect heightened sympathetic activation, reduced oxygen delivery, or other physiological burdens before overt symptoms become apparent [1]. Recognizing these changes in real time enables divers or supervisors to modify dive plans, initiate controlled ascents, or terminate dives to prevent adverse outcomes. In addition, when HR and SpO_2_ data are shared with a dive buddy or surface supervisor, timely intervention becomes possible even if the diver is unable to respond independently. Such proactive safety responses are especially important during deep, cold, or repetitive dives, where physiological reserves may already be diminished [7].

### 5.2. Practical Applications and Integration in Safety Systems

Recent advances in wearable sensing and diving-specific monitoring platforms have led to the development of integrated safety systems aimed at reducing underwater risks [6,7]. Customized diving computers that combine biometric sensing (e.g., HR and respiration) with environmental data (depth, pressure, temperature) have been designed to support diver decision-making and improve operational safety [6]. These systems process physiological data in real time and can display context-specific warnings such as ascent-rate violations, hypothermia risk, or abnormal heart rate responses. Parallel research has explored frameworks for wearable ECG monitoring with emergency alert functions during SCUBA diving. In prototype evaluations, real-time ECG signals were transmitted to mobile devices in waterproof housings, and vibration alerts were issued when predefined thresholds were exceeded, demonstrating feasibility for early detection of cardiovascular strain [5].

Integration into broader safety systems is also being considered. Some proposals link wearable sensors to diver-log management platforms and surface-based monitoring stations, allowing supervisors to track real-time biometrics and environmental conditions simultaneously [6,7]. In the future, such systems could interface with Human-Robot Interaction (HRI) frameworks to enable automated support or rescue deployment when abnormal patterns are detected, as suggested in HRI-focused diver safety equipment studies (e.g., emergency beacons, diver-robot communication) [7]. Collectively, these developments illustrate how wearable sensors can move beyond standalone monitoring to form part of a comprehensive safety ecosystem, supporting both individual divers and supervisory teams in preventing accidents and enabling timely intervention.

## 6. Future Directions

### 6.1. Development of Integrated Multisignal Monitoring

**Figure 2:**
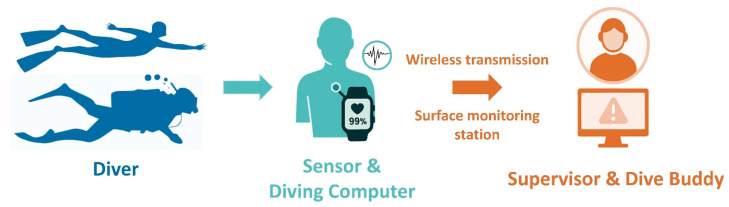
Conceptual design of an integrated underwater physiological monitoring and alert system. Conceptual design of an integrated underwater physiological monitoring and alert system. Wearable sensors placed on the diver (e.g., chest, forehead, cheek) measure heart rate and peripheral oxygen saturation and transmit data to a diving computer. Data are then transmitted wirelessly to a surface monitoring station, allowing supervisors and dive buddies to receive real-time information and alerts to support timely intervention.

Building on current HR and SpO_2_ monitoring platforms, future work should focus on integrating additional physiological signals to create a more comprehensive understanding of diver status [7]. Beyond HR and SpO_2_, signals such as HR variability, respiratory patterns, skin temperature, and even muscular activity might be explored as potential indicators of autonomic regulation and workload. For instance, abrupt autonomic changes preceding panic or hyperventilation may not be fully captured by HR and SpO_2_ alone. Pupillary responses, which reflect sympathetic activity and psychological stress, also represent a promising biomarker, and recent research on infrared-based underwater pupillometry suggests that integrating these data with HR–SpO_2_ readings could enable predictive modeling of panic episodes. To support these applications, future wearable systems may integrate multiple sensor types, including ECG modules for cardiac activity, forehead- or temple-mounted SpO_2_ sensors, inertial measurement units (IMUs) for motion tracking, and depth/pressure and temperature gauges for environmental monitoring [5]. Textile-based respiration and skin-temperature sensors embedded in diving suits also represent a promising direction for continuous, non-invasive measurement [6]. In terms of communication, underwater data transfer may rely on acoustic telemetry, short-range optical signaling, or onboard storage with post-dive analysis, since conventional Bluetooth is limited in submerged environments [7]. These multimodal systems would require robust waterproof sensor arrays and advanced signal fusion techniques to ensure accuracy in highly dynamic underwater environments. The development of such integrated devices could ultimately lead to more reliable assessments of diver fatigue, thermal stress, and early signs of physiological instability. However, beyond simply expanding the range of monitored signals, achieving truly reliable measurements will also require hardware and algorithmic improvements. Examples include waterproof housings with enhanced pressure resistance, motion-resistant electrodes, and oximetry designs that compensate for cold-induced vasoconstriction, as well as adaptive filtering and signal-quality indexing to reduce artifacts. These combined advances could substantially improve the robustness and reliability of underwater physiological monitoring. A conceptual overview of a future integrated monitoring and alert system is shown in Figure 2.

### 6.2. Algorithm-Based Early Warning Systems

Most current monitoring devices rely on predefined thresholds to trigger alerts, which may not account for individual variability or environmental complexity [7]. Future research should emphasize algorithm-based approaches, leveraging machine learning and adaptive models to interpret continuous data streams in real time. For example, real-time ECG analysis with QRS (Q, R, and S wave complex) detection algorithms and automated alert systems has already shown feasibility for underwater monitoring [5], while decision-tree methods such as Classification and Regression Trees (CART) have been applied to identify abnormal respiration and temperature patterns [6]. More recently, prototype diver masks equipped with AR/HUD displays have explored AI-assisted evaluation of consciousness and physiological stability [7]. By learning a diver’s baseline patterns and detecting deviations, these systems could reduce false alarms while providing earlier and more personalized warnings for hypoxic stress, excessive workload, or other adverse conditions. Implementing predictive analytics would represent a shift from simple monitoring to proactive safety management.

### 6.3. Recommendations for Future Study Designs

To validate and refine these emerging technologies, large-scale field studies are needed in real open-water environments. Most existing data are derived from controlled laboratory settings or small pilot trials, which may not fully capture the variability encountered in recreational, industrial, or military diving operations [5,6,7,43]. Future studies should include diverse diver populations, varied depths and durations, and operational stressors such as cold water or repetitive dives. In particular, it will be important to account for inter-individual variability, since physiological responses may differ according to age, physical fitness, diving experience, or chronic health conditions. Subgroup analyses could help to define safer ranges for specific populations, while AI-based approaches may enable personalized thresholds by learning an individual diver’s baseline and detecting deviations in real time. By integrating continuous physiological data such as HR and SpO_2_ with traditional dive profiles, these wearable systems may enable earlier detection of hypoxic stress, contribute to reducing the risk of decompression sickness, and ensure continuous tracking even in hostile underwater environments. Collaborative research between academic institutions, equipment manufacturers, and dive safety organizations could accelerate progress toward reliable, field-tested systems that support both individual divers and supervisory teams.

### 6.4. Applications in Special Populations

Scuba diving induces physiological changes such as bradycardia, central blood volume shift, and increased oxygen availability. In individuals with stable cardiovascular conditions, these effects may offer therapeutic potential by reducing myocardial workload and supporting autonomic balance. Controlled diving under safe conditions could function as a form of low-intensity aerobic exercise. Some preliminary observations suggest possible benefits, including improved mood, reduced pain, and enhanced adherence to physical activity [57,58]. However, due to risks like arrhythmias or barotrauma, careful screening and supervision are essential [48]. Further research is needed to explore the safety and feasibility of diving-based rehabilitation in clinical populations. It should also be recognized that age, physical fitness, and comorbid conditions may markedly influence cardiovascular and oxygenation responses in these populations, underscoring the importance of individualized screening and tailored monitoring approaches.

## 7. Conclusions

This review synthesized current knowledge on HR and SpO_2_ as key physiological indicators in underwater environments. These parameters provide valuable information on a diver’s adaptive responses, including early signs of hypoxic stress and overall cardiopulmonary demands. Although recent developments in wearable monitoring devices and prototype safety systems have shown promising potential, most existing studies remain limited to laboratory or simulated conditions, and significant technical challenges such as signal reliability, waterproofing, and long-term usability still need to be addressed.

Looking ahead, future progress will require the development of integrated multisignal monitoring platforms, the application of algorithm-based early warning systems, and large-scale validation in real open-water settings. By closing these gaps, the diving research community can move beyond isolated measurements toward a comprehensive, real-time understanding of physiological responses under water. Ultimately, advancing these monitoring technologies will support the overarching goal outlined in this review: enhancing diver safety and driving innovation in the study of human physiology in challenging underwater environments. Beyond recreational diving, such advances also hold broader relevance for occupational safety in commercial diving operations, military missions requiring sustained underwater activity, and deep-sea research expeditions. Framing wearable monitoring in these wider contexts underscores its potential not only as a tool for sports and exploration but also as an emerging component of healthcare and human performance management in extreme environments. At the same time, cost and accessibility remain practical challenges, as specialized waterproof and pressure-resistant devices are often expensive and not widely available to recreational divers, underscoring the need for future innovations that balance technical performance with affordability and broader dissemination.

## Figures and Tables

**Table 1:** Summary of Key Studies on Diver Physiological Monitoring.

Author (Year)	Participants	Environment	Measured Parameters	Main Findings
Flouris and Scott (2009) [12]	3 trained scuba divers	Pool (5 m, 27 °C)	HR, HRV	HR increased during challenging task performance; HRV decreased.
Marabotti et al. (2013) [31]	18 experienced scuba divers	Pool (5 m, 10 m, 21 °C)	HR	Post-dive HR significantly decreased; no marked bradycardia during dive. Left ventricular functional changes observed.
Bosco et al. (2014) [42]	16 trained scuba divers	Sea (various temperatures)	HR, ECG	High-quality 12-lead ECG recording feasible underwater. HR increased before diving, decreased during diving, and decreased further after diving. Arrhythmias and conduction abnormalities observed but no evidence of ischemia.
Marongiu et al. (2015) [4]	7 trained freedivers	Sea (10 m, 20 m, 30 m, 22–25 °C)	HR, SpO_2_	HR and cardiac output increased during descent/ascent. HR decreased during static apnea. SaO_2_ decreased with depth.
Zenske et al. (2020) [26]	3 experienced scuba divers	Lake (5–10 °C)	HR, HRV	HR decreased during dive, transient HR increase immediately after incident. HRV indices increased overall.
Schaller et al. (2021) [1]	13 scuba divers with varying experience	Lake (5–20 °C)	HR, HRV	HR high before dive, decreased post-dive. Autonomic conflict observed.
Mulder et al. (2021) [43]	6 freedivers	Pool (32 °C)	HR, SpO_2_	SUB oximeter matched commercial devices. Forehead sensor more reliable than finger under vasoconstriction.
Mulder et al. (2021) [13]	4 elite freedivers	Sea (19 m and 73 m, 22 °C)	HR, SpO_2_	HR and SpO_2_ monitored down to 82 m. Greater SpO_2_ desaturation at depth. HR increased pre-dive, decreased during descent, peaked after ascent.
Bruzzi et al. (2022) [29]	4 Brazilian Navy scuba divers	Sea (Antarctica, −1.7 °C)	HRV	Short dives reduced both parasympathetic and sympathetic activity (HRV indices).
Mulder et al. (2023) [27]	6 recreational freedivers	Pool (11 m, 32 °C)	HR, SpO_2_	Progressive SpO_2_ decline during repeated dives; some divers reached SpO_2_ as low as 47%. HR increased before diving, decreased during dive, and recovered post-dive.
Di Pumpo et al. (2023) [11]	1 non-professional scuba diver	Pool (15 m)	HR, SpO_2_	SpO_2_ and ORi reflected oxygen status. Hyperoxia identified at depth. HR monitoring basic.
Mulder et al. (2023) [28]	14 elite freedivers	Sea (10–25 m shallow, >35 m deep, 22 °C)	HR, SpO_2_	Greater SpO_2_ desaturation in deep dives, HR higher than shallow dives, autonomic conflict observed.

HR: Heart rate; HRV: Heart rate variability; ECG: Electrocardiogram; SpO_2_: Peripheral oxygen saturation; SaO_2_: Arterial oxygen saturation; ORi: Oxygen reserve index.

**Table 2:** Maximum Operating Depths Based on Oxygen Fraction (FiO_2_) to Avoid CNS Oxygen Toxicity (PO_2_ ≤ 1.6 ATA).

O_2_ Fraction (FiO_2_)	Maximum Depth (m) for 1.6 ATA PO_2_
21% (Air)	66 m
32% (Enriched Air Nitrox 32%)	33.8 m
36% (Enriched Air Nitrox 36%)	28.4 m
100% O_2_	6.0 m

FiO_2_, fraction of inspired oxygen; PO_2_, partial pressure of oxygen; ATA, atmospheres absolute. Maximum depths at which the inspired oxygen partial pressure (PO_2_) reaches 1.6 ATA, according to different oxygen fractions in breathing gas mixtures. Depths are calculated using the equation: Depth (m) = [(PO_2_/FiO_2_) − 1] × 10.

**Table 3:** Proposed SpO_2_ Alert Thresholds by Depth (Air Breathing).

Depth	Pressure (ATA)	Expected SpO_2_	Warning Threshold
Surface (0 m)	1.0	95–99%	<94%
10 m	2.0	97–100%	<96%
20 m	3.0	98–100%	<97.5%
30 m	4.0	~99–100%	<98.5%

SpO_2_: Peripheral oxygen saturation; ATA: Atmospheres absolute.

## Data Availability

No new data were created or analyzed in this study. Data sharing is not applicable to this article.

## Abbreviations

The following abbreviations are used in this manuscript:

HR	Heart Rate
SpO_2_	Peripheral Oxygen Saturation
DCS	Decompression Sickness
MDR	Mammalian Diving Reflex
ANS	Autonomic Nervous System
HRV	Heart Rate Variability
ECG	Electrocardiogram
SaO_2_	Arterial Oxygen Saturation
ORi	Oxygen Reserve Index
PiO_2_	Partial Pressure of Inspired Oxygen
FiO_2_	Fraction of Inspired Oxygen
PO_2_	Partial Pressure of Oxygen
ATA	Atmospheres Absolute
PaO_2_	Arterial Partial Pressure of Oxygen
HRI	Human-Robot Interaction
